# Current Use of Equine Laparoscopy in Urogenital Disorders: A Scoping Review of the Literature from 2000 to 2021

**DOI:** 10.3390/vetsci9020041

**Published:** 2022-01-22

**Authors:** Paola Straticò, Giulia Guerri, Adriana Palozzo, Vincenzo Varasano, Lucio Petrizzi

**Affiliations:** Veterinary Teaching Hospital, Faculty of Veterinary Medicine, University of Teramo, Località Piano D’Accio, 64100 Teramo, Italy; pstratico@unite.it (P.S.); gguerri@unite.it (G.G.); vvarasano@unite.it (V.V.); lpetrizzi@unite.it (L.P.)

**Keywords:** laparoscopy, urogenital disorders, surgery, horse, mule, donkey

## Abstract

(1) Background: Laparoscopic surgery replaced traditional invasive techniques for the treatment of common urogenital disorders in equids. The aim of this review is to evaluate applications and the development of urogenital laparoscopy from 2001 to 2021. (2) Methods: A scoping review of literature was undertaken according to the Preferred Reporting Items for Systematic Reviews and Meta-Analysis (PRISMA) guidelines for scoping reviews on three databases (NCBI-PubMed, Web of Science-Thompson Reuters, and SciVerse Scopus). (3) Results: A total of 452 papers were identified. After duplicate removal and title screening, 181 papers underwent abstract screening. Of these, 160 + 10 papers (cited by others) were assessed for eligibility according to the PICOs. A total of 132 papers were considered eligible. Most of the research was focused on ovaries and testes, followed by urinary bladder and general articles about laparoscopy in horses. We identified 43 original studies (33%, RCT, NoRCT, and experimental trials), 39 case series/retrospective studies (29%), 37 case reports (28%), and 13 reviews (10%, narrative or systematic). (4) Conclusions: Gonadal disorders were the most investigated. Hand-Assisted Laparoscopic Surgery (HALS) and laparoscopic-assisted surgery represent valuable options for more challenging conditions (uterine and urinary bladder disorders).

## 1. Introduction

Urogenital disorders in equids occur due to a variety of conditions of different organs. Abdominal urinary pathologies are mainly represented by urolithiasis [1,2] and bladder neoplasia [3,4,5,6,7,8], whereas genital disorders are usually related to testes retention [9,10,11,12], ovarian neoplasia [13], or unwanted behavior in the mares [14]. Little research has involved uterine conditions mainly for resolution of uterine tears, neoplasia, or infections [15].

Until laparoscopy was first introduced in equine surgery in 1986, the approaches to the aforementioned disorders were typically laparotomic through median or paramedian accesses [16]. From then on, laparoscopic research has focused on techniques based on new instrumentations, combining open and mini-invasive approaches, different positioning of the horse, protocols for standing sedation, methods used to provide hemostasis, and different position, and number of the portals.

Laparoscopy has well-known advantages over laparotomy: an excellent visualization of the abdominal cavity, better assessment of hemostasis, smaller body wall incisions, and secondary reduced risks of wound infection, associated with faster recovery. Moreover, the possibility to avoid general anesthesia, together with its related risk and costs, makes laparoscopy the best surgical option in many conditions. On the other hand, the availability of the instrumentation, the cost related to its maintenance, and the surgeon’s familiarity with the technique limits its use. As a rule, the decision for laparoscopy relies on equipment availability, the type of disorder, the patient’s characteristics, and the surgeon’s ability.

Differently from systematic reviews which focus on a specific question, a scoping review has as the main goal to identify and categorize the evidence about a subject [17]. Similarly, they follow a systematic approach to study selection, limiting the bias that exists when a narrative review is undertaken, thus providing a transparent and replicable means of describing the literature on a topic.

The aim of this scoping review was to investigate the equine urogenital disorders most treated with laparoscopy from years 2001 to 2021 and how the research in this field was conducted in the last 20 years.

## 2. Materials and Methods

This scoping review was conducted according to the Preferred Reporting Items for Systematic Reviews and Meta-Analysis (PRISMA) guidelines for scoping reviews [18]. The PRISMA checklist containing information relevant to this scoping review is reported in Appendix A.

Three major veterinary databases were used between August and November 2021 to obtain a list of peer-reviewed publications: NCBI-PubMed, Web of Science-Thompson Reuters, and SciVerse Scopus. Additional relevant records were identified through the references of retrieved publications.

To reduce limitations on publications, only language filters for the type of study were applied: only English language peer-reviewed papers published between 2001 and 2021 were considered. All databases were searched using the advanced search function. The last research was conducted on 20 November 2021.

For the NCBI PubMed database the search string was the following:

(((((horse) OR (mule)) OR (donkey)))) AND (laparoscopy)) AND (fft[Filter]),,”Full text, from 2001 2021”,”((““horse s”“[All Fields] OR ““horses”“[MeSH Terms] OR ““horses”“[All Fields] OR” “horse”“[All Fields] OR (““equidae”“[MeSH Terms] OR ““equidae”“[All Fields] OR ““mule”“[All”“donkey”“[All Fields] OR ““donkeys”“[All Fields])) AND (““laparoscopy”“[All Fields] OR ““laparoscopy”“[MeSH Terms] OR ““laparoscopy”“[All Fields] OR ““laparoscopies”“[All Fields]) AND ““loattrfull text”“[Filter]) AND ((fft[Filter]) AND (2001:2021[pdat]))”,186,05:41:02.

For the Web of Science database, the search string was the following:

laparoscopy (All Fields) and horse (All Fields) and 2001–2021 (Year Published) not gastrointestinal (All Fields)

For the SciVerse Scopus database the search was the following:

laparoscopy AND horse AND urogenital AND (EXCLUDE (PUBYEAR, 2000 OR EXCLUDE (PUBYEAR, 1999) OR EXCLUDE (PUBYEAR, 1998) OR EXCLUDE (PUBYEAR, 1997) OR EXCLUDE (PUBYEAR, 1996) OR EXCLUDE (PUBYEAR, 1988)) AND (LIMIT-TO (DOCTYPE, “ar”) OR LIMIT-TO (DOCTYPE, “re”)) AND (LIMIT TO (SUBJAREA, “VETE”)) AND (LIMIT-TO (LANGUAGE, “English”)) AND (LIMIT-TO (SRCTYPE, “j”))

laparoscopy AND donkey AND urogenital AND (LIMIT-TO (DOCTYPE, “ar”)) AND (LIMIT-TO (SUBJAREA, “VETE”)) AND (LIMIT-TO (LANGUAGE, “English”)) AND (LIMIT-TO (SRCTYPE, “j”))

laparoscopy AND mule AND urogenital AND (LIMIT-TO (SUBJAREA, “VETE”)) AND (LIMIT-TO (LANGUAGE, “English”)) AND (LIMIT-TO (SRCTYPE, “j”))

Editorials, proceedings, meeting abstracts were not included.

All merged publications were imported into Mendeley reference management and social citation sharing software. Duplicates were removed using the duplicate screening process of the software, and manually when minor differences could not be detected by the software itself. Once duplicates were removed, titles and abstracts were manually screened for relevance.

The first stage of screening was made reviewing only the title and abstract of the paper. If it was unclear whether a citation did or did not meet the inclusion criteria at this step, the paper was included in the second stage of screening, which dealt with full-text screening for eligibility of the included papers. Eligibility was assessed following the objectives modified from “PICOs”: Population: horses receiving surgery at the urogenital tract; Intervention: laparoscopy; Outcome: postoperative recovery from the disorder. Additionally, reviews about standing urogenital surgery and laparoscopy were included.

Full text papers were accessed from university libraries, library journal subscriptions, and open access sources. Those papers that could not be retrieved and could not undergo the second stage screening were removed.

Categorization of each paper was made on electronic sheets (Microsoft Excel 16.54) and included authors, country of origin, aim of the study, publication type [narrative or systematic reviews, original studies (randomized and non-randomized controlled trials-RCT/NoRCT, experimental studies), case reports, case series/retrospective studies], target organ, and type of surgery.

## 3. Results

### 3.1. Selection of Sources of Evidence

The total number of papers that were retrieved was 452, which was divided as follows:

- NCBI-PubMed: 186.
- Web of Science-Thompson Reuters: 158.
- SciVerse Scopus: 108.

After removal of duplicates (*n* = 61) and non-applicable titles (human studies, non-equid patients) (*n* = 210), 181 abstracts underwent the first stage of screening. Those papers that were not considered eligible according to the PICOs (n. 21), were removed.

**Figure 1:**
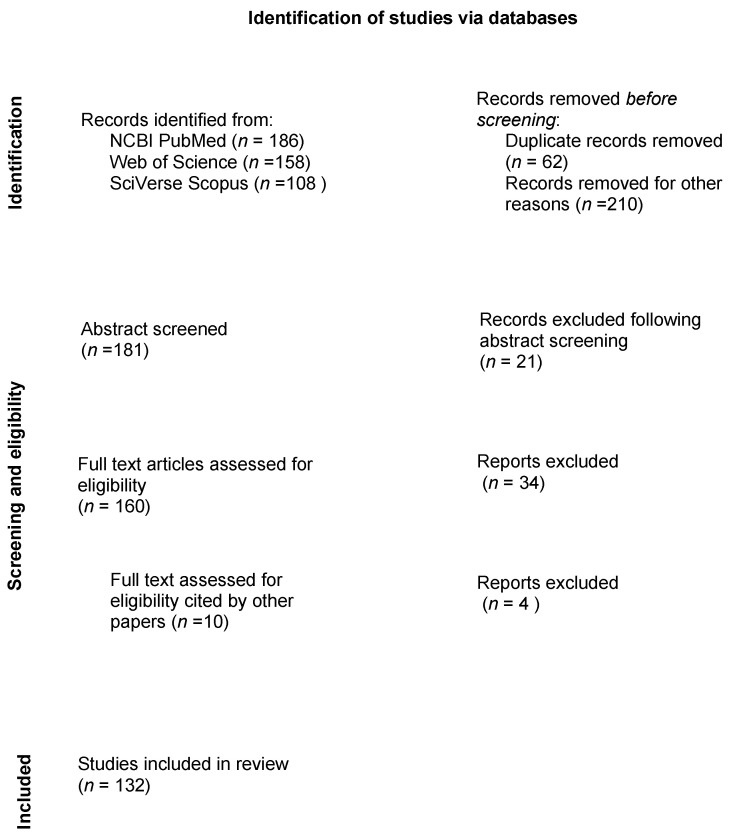
Flow diagram of the search strategy for a scoping review in equine urogenital laparoscopy. Number of publications that were reviewed and excluded during each step of the review process.

A total of 160 papers underwent the second stage of screening considering the full paper version of the studies (Figure 1). During full paper screening, 10 new papers were included for eligibility, as cited by others, for a total number of 170 full papers undergoing eligibility screening. Of these, 38 (22%) were removed because they were not adherent to the PICOs principles. After the second stage of screening, 132 papers were included as eligible (78%) [19,20,21,22,23,24,25,26,27,28,29,30,31,32,33,34,35,36,37,38,39,40,41,42,43,44,45,46,47,48,49,50,51,52,53,54,55,56,57,58,59,60,61,62,63,64,65,66,67,68,69,70,71,72,73,74,75,76,77,78,79,80,81,82,83,84,85,86,87,88,89,90,91,92,93,94,95,96,97,98,99,100,101,102,103,104,105,106,107,108,109,110,111,112,113,114,115,116,117,118,119,120,121,122,123,124,125,126,127,128,129,130,131,132,133,134,135,136,137,138,139,140,141,142,143,144,145,146,147,148].

### 3.2. Synthesis of Results

**Figure 2:**
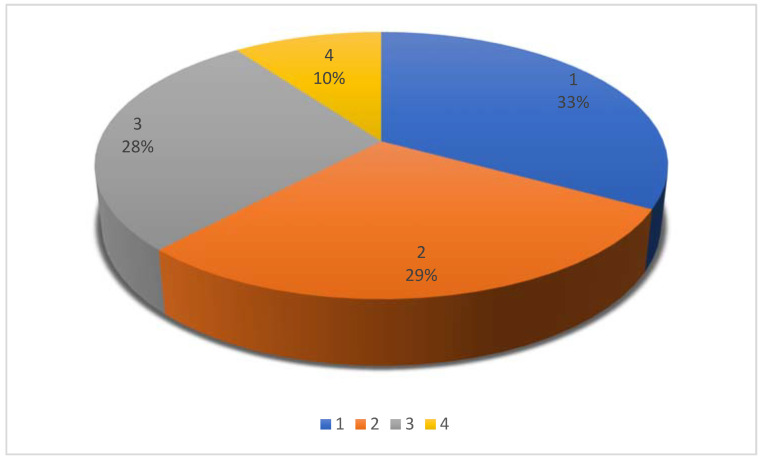
Pie chart showing publication type for 132 citations included in a scoping review (1: original studies 33%; 2: case series/retrospective studies 29%, 3: case reports 28%; 4: reviews 10%).

We identified 43/132 original studies, 39/132 case series/retrospective studies, 37/132 case reports, and 13/132 reviews (Table S1) (Figure 2).

**Figure 3:**
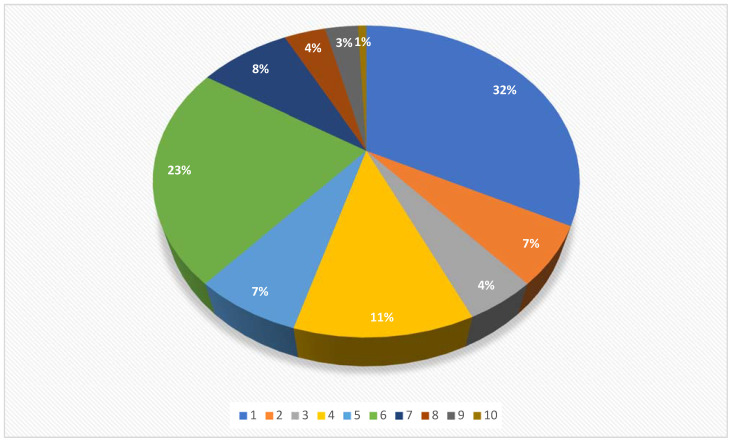
Pie chart showing the distribution of the 132 citations included in a scoping review, categorized according to the target organ/structure (1: ovaries 32%, 2: uterus 7%, 3: ovaries-uterus 4%, 4: urinary bladder 11%, 5: inguinal rings 7%, 6: testes 23%, 7: general information about laparoscopy 8%, 8: kidney 4%, 9: uterine tubes 3%, and 10: ductus deferens 1%).

When the target organ was considered, the identified research focused mainly on ovaries (42/132) and testes (30/132), followed by urinary bladder (15/132), general information about laparoscopic surgery (11/132), inguinal rings (9/132), uterus (9/132), ovaries- uterus (6/132), kidney (5/132), uterine tubes (4/132), and ductus deferens (1/132) (Figure 3).

**Figure 4:**
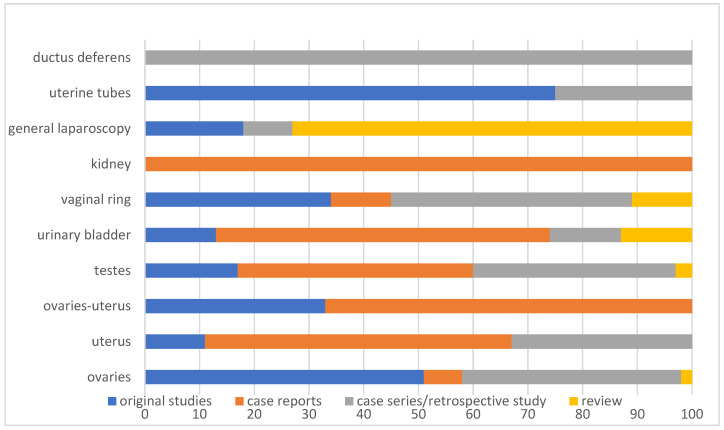
Horizontal bar chart showing the publication type for 132 citations included in a scoping review, categorized according to the target organ/structure and expressed as a percentage (%).

Organ-related research was distributed as follows (Figure 4):

Ovaries:

4/42 (10%) case reports.

1/42 (2%) review.

17/42 (40%) case series/retrospective studies.

20/42 (48%) original studies.

Testes:

5/30 (17%) original studies.

11/30 (37%) case series/retrospective studies.

13/30 (43%) case reports.

1/30 (3%) review.

Urinary bladder:

9/15 (60%) case reports.

2/15 (13%) reviews.

2/15 (13%) case series/retrospective studies.

2/15 (13%) original studies.

General information about laparoscopic surgery

8/11 (73%) reviews.

1/11 (9%) case series/retrospective study.

2/11 (18%) original studies.

Uterus:

3/9 (33%) case series/retrospective studies.

5/9 (56%) case reports.

1/9 (11%) original study.

Inguinal rings:

3/9 (33%) original studies.

4/9 (44%) case series/retrospective studies.

1/9 (11%) case report.

1/9 (11%) review.

Ovaries and uterus:

4/6 (67%) case reports.

2/6 (33%) original studies.

Kidneys:

5/5 (100%) case reports.

Uterine tubes:

1/4 (25%) case series/retrospective study.

3/4 (75%) original studies.

Ductus deferens:

1/1 (100%) case series.

## 4. Discussion

This scoping review was meant to investigate the equine urogenital disorders that were most treated with laparoscopy from 2001 to 2021. We also tried to outline the type of studies according to the target organ and/or region.

The use of three main databases allowed a comprehensive search of all the potentially relevant literature: over the last 20 years, it was possible to identify 132 manuscripts that were eligible to be included in this scoping review.

Few reviews in equine laparoscopy were published from 2001 to 2021, mainly represented by narrative reviews about general abdominal surgery [130], the use of laparoscopy [68,107,129], or about standing surgical procedures [19,54,63].

Reviews focused on ovariectomy [67,87], cryptorchidectomy [67,76], management of urolithiasis with reference to laparoscopic approaches [53,61], or standing urogenital surgery [127]. To the authors’ knowledge, no scoping review about urogenital laparoscopic surgery has been published before.

A scoping review tries to identify evidence about a subject, rather than evaluate the quality of research. For this reason, the quality assessment tool (QUIPS) developed by Hayden et al. 2013 [149] was not used. Since QUIPS is considered valuable to evaluate the risk of bias and the quality of eligible papers, the lack of these data could be considered a limitation of the study but considering the risk of bias and quality of research went beyond our goals. Moreover, we used a single individual approach to the research, leading to a potential bias of the results.

The most investigated organ was the ovary, with 42/132 of publications (32%), followed by the testes (30/132, 23%). Ovariectomy and cryptorchidectomy have always been a challenge in equine surgery due to the limited access and visualization with a laparotomic approach, and to the highest request for surgical intervention. During the last 20 years studies on standing flank laparoscopy, as first described by Fischer et al. [16], have mainly focused on managing a variety of conditions [29,32,38,40,75,79,85,98,104,111,132,138] that were previously treated under general anesthesia and developing new methods for hemostasis [23,27,33,52,55,64,65,71,72,86,112,116,126,128,131]. Many publications tried to suggest alternative methods to manage enlarged ovaries [41,83,89] and abdominally retained testes [124] and reduce surgical time, decrease damage to the abdominal wall during extraction and avoid secondary complications related to the incision, as well as reduce the risk of dropping the gonad within the abdominal cavity [41]. On this topic, the use of morcellator is without any doubt the most investigated aspect, although carrying some disadvantages (high costs of the equipment and risk of damage to other organs) [41,83,89,124]. 

Recently, a single side laparoscopic approach for bilateral laparoscopic ovariectomy has been described [35,49]. Its main advantage is the possibility to enter the abdominal cavity from the same flank through five portals, avoiding bilateral paralumbar incisions. On the other hand, this approach is not suitable for larger mares. Moreover, one of the portals must be localized between the 17th and 18th ribs, with the risk of thoracic cavity penetration [150].

A new approach to the abdominal cavity for treating ovarian disorders is represented by the transvaginal natural orifice transluminal endoscopic surgery [21,96,97] (NOTES). This was first introduced in swine [151,152] and then used on animals and people [153,154,155]. It can be considered a development of abdominal surgery via transvaginal colpotomy that is well known in mares [133,136,156,157,158]. Advantages of NOTES over laparoscopy are mainly represented by the lack of abdominal wounds, but concerns are expressed about the potential transvaginal abdominal contamination and possibility to develop adhesion at the entry site. Moreover, long instruments are mandatory, and the procedure is considered more technically challenging than laparoscopy [21].

Laparoscopic ovariectomy was also used to treat unwanted estrus-related behavior in equids, with conventional approaches and follow-up with the owners to investigate the success of removal on behavior [36,50,100]. Devick et al. (2020) [35] investigated a relationship between owners’ satisfaction after bilateral ovariectomy toward complained behavior and endocrinological profiles of the mares. They concluded that no correlation could be found, and that the reason for the complaint must include ovarian neoplasia (early granulosa cell tumors). According to Collar and colleagues no association could be found even between behavior and response to altrenogest in mares [36]. Even if mostly unable to maintain a pregnancy, this aspect was investigated also in mule mare, with similar results [100]. In all cases, client perspective was used to assess behavioral improvement, which could have led to bias.

HALS (Hand-Assisted-Laparoscopic-Surgery) proved to be a valid option in the field of equine laparoscopy as an aid in case of particularly enlarged organs or neoplasia [26,69,92], especially when the size of the mass cannot be reduced intraabdominally [41,74,102,117,119,142]. These techniques provide excellent visualization of the abdominal cavity, while the direct manipulation of the target organ with the surgeon’s hand allows more efficient maneuvers than instrumental control. Compared to standard laparoscopy, HALS is much easier to learn, thanks to the three-dimensional feedback of one hand into the surgical field that can be used for blunt dissection, as a retractor, or as an aid in providing hemostasis [60].

In the two last decades, novel interest in the field of urogenital laparoscopic surgery is shown in equids different from the horse, mainly for gonadal disorders [25,99,100]. Pepe et al. (2005) [99] described the use of an endoscopic linear stapler to transect the spermatic cord under laparoscopic guidance, leading to testicular avascular necrosis in six weeks without testicle removal in asses. Testosterone follow-up was available for 12 months, during which no signs of revascularization were observed (testosterone <15 ng/dL). A custom-made device for isolation, coagulation, and cutting of the mesovarium was described in donkeys [25]. The main advantage of this device was a triple function in one instrument, although a considerable amount of smoke was produced.

Surgery of the urinary bladder (15/132, 11%) can be considered a wide chapter mainly dedicated to the treatment of urolithiasis [88,93,113,134,141], neoplasia [4], and ruptures [91,95,108,109,123,137]. Although thorough laparoscopic approaches to the urinary bladder were described [159], many surgeons seem to prefer a combination among laparoscopic and laparotomic techniques, to obtain a better visualization of the urinary bladder under laparoscopic guidance and avoid the need for intracorporeal sutures exteriorizing the organ. Laparoscopy carries the advantage of reducing tension on the wall of the bladder and the avoidance of larger incisions through the abdominal wall with the risk of wound complication or vessel damage [53,160,161]. On the other hand, the increased probability of urine contamination within the abdomen and calculi fragmentation into the peritoneal cavity make the technique particularly challenging. For these reasons, in urinary bladder surgery, the choice of the specific technique is based on the patient’s gender, surgeon’s preference, and urolith diameter.

A valuable aid for the synthesis of the bladder is represented by barbed sutures (mono-or bidirectional), which avoid the need of intracorporeal knots. Although already used in human bladder surgery [162,163,164,165], few studies are published in vivo on equine patients [37,103,166]. Only ex-vivo studies are reported in equine urogenital disorders [91,123], showing equal efficacy for both types of sutures (mono and bidirectional) during experimental cystorrhaphies, although the surgeon’s confidence with the technique is mandatory to ensure a good result. Since in human studies, these knotless sutures decreased the amount of suture material incorporated into the closure, as well as surgical time, ensuring the same performances, further research in veterinary surgery should be undertaken in vivo.

Uterine disorders (9/132, 7%) considered in literature were neoplasia [74,94,102], tears [26,43,51,92], infections [43,148], and ligament laxity [28,37].

Ovariohysterectomy (6/132, 4%) was also described thorough a laparoscopic approach although laparoscopic-assisted approaches were generally preferred: after laparoscopic hemostasis and resection of mesovarium and mesometrium in the standing mare, surgery continues with an open withdrawal of the uterus in dorsal recumbency under general anesthesia [26,44,46], or a transvaginal approach under sedation [59], or unilateral hand-assisted removal [79,148]. The transvaginal uterine removal is described in a case report (of uterine adenocarcinoma) that was particularly challenging because of the uterine tone. Moreover, the authors suggest uterine lavage prior to surgery, to reduce the risks of abdominal contamination in case of rupture. Since no objective evaluation of post-operative pain was accomplished, no conclusion about the advantages of this technique can be drawn.

Inguinal hernia repair (9/132, 7%) was often attempted and described in stallions with a therapeutic and/or prophylactic purpose, aiming at preserving the testicular vascularization and function. Intracorporeal suture closure of the internal inguinal rings [30], mesh or cyanoacrylate application at the same site [121], and peritoneal flaps [62,103,146,147] are all surgical options that were considered for inguinal hernias. A literature review was published investigating the laparoscopic closure of the inguinal rings in stallions [61]. All these techniques are described in a standing laparoscopic approach. Different intra and post-operative complications were described for all the techniques (bleeding during peritoneal flaps, reherniation, testicular atrophy, hemospermia, and scrotal edema). The authors conclude that for unknown reasons peritoneal flaps are associated with the most severe postoperative complications. Further research is requested to assess the postsurgical testicular function.

Renal disorders amenable to surgical treatment are relatively uncommon in horses. Hand-assisted laparoscopic nephrectomy was described [80] and used for treating unresolved ureteral ectopia in a foal [34], renal disease [114], or neoplasia [69,119]. Compared to laparotomy, laparoscopic nephrectomy avoids general anesthesia and rib resection with good visualization and secure ligation of renal vessels. The hand-assisted technique does not avoid the need for a mini-laparotomy but simplifies the procedure, reduces surgical time, and allows more direct and safer circumferential isolation and mobilization of the kidney [114].

In some reports, assessment, and treatment of oviductal patency in mares under laparoscopic guidance was also faced, with good results (4/132 3%) [22,24,82,101]. The use of fluorescent beads laid down into the infundibulum under laparoscopic guidance, allowed the evaluation of plugs in the oviducts of mares and the shape of the infundibulum; obstruction of the oviduct and rough shape of the infundibulum are two potential causes of potential infertility [24] that could be addressed, although peritonitis and bowel puncture were described as potential risks. PGE_2_ was successfully used to restore oviductal patency as a topical gel application over the tube under laparoscopic guidance [22].

Only 1 study out of 132 (1%) was focused on laparoscopic vasectomy in four stallions with a left laparoscopic flank approach, achieving an adequate azoospermia without interference with the reproductive behavior. The standing procedure was faster and easier than the same performed under general anesthesia, without the need for specialized equipment. Nevertheless, the availability of a vessel sealing device facilitates the surgery [140].

Although we did not include publications focused on the protocols for general anesthesia and sedation, a great interest was shown by authors on this topic [167,168,169,170,171,172]. Caudal epidural anesthesia with alpha-2 agonist and opioid combined with sedation, was a great support in providing analgesia during standing procedures [71,173,174,175,176], reducing surgical times and risks related to the patient and to the equipment. Considerations about the animal size must be done, since the drugs given epidurally should reach the emergence of the ovarian nerve plexus at the caudal mesenteric ganglion, ventral to the lumbar spinal vertebra 3 (L3) [177]. Moreover, local anesthetic techniques were investigated to reduce pain during the laparoscopic procedures and to minimize the amount of sedative and anesthetic drugs to be used [81]. Among them, local infiltration of lidocaine in the mesorchium/mesovarium or gonadal parenchyma were compared, with the best results for intramesovaric inoculation rather than intraovarian [57]. Moreover, attention was given to the effects of pneumoperitoneum on physiologic variables [178,179,180].

Due to the breadth of the literature included and to the different procedures described a comparison among studies was not carried out [131]. Usually, comparisons are impaired because of different definitions of intra- and post-operative complications as defined by the authors and because of the different architecture of the studies designs.

## 5. Conclusions

Laparoscopic urogenital surgery has a variety of applications mostly related to the treatment of gonadal disorders. The development of HALS allowed the diffusion of the mini-invasive approaches also to more challenging conditions, such as uterine and renal pathologies. The urinary bladder is usually approached with laparoscopic-assisted methods, rather than thorough laparoscopic method.

Although the advantages of laparoscopy over laparotomy are well known, the decision-making process takes into consideration patients characteristics (size and temperament), equipment availability, clinical condition (mainly the size of the mass to remove), and the surgeon’s preference and confidence with mini-invasive techniques.

Nevertheless, in the authors’ opinion, a comparison between studies is difficult because of the variety of designs and case reports. More RCT should be designed to compare clinical conditions and treatments.

## Supplementary Materials

The following supporting information can be downloaded at: https://www.mdpi.com/article/10.3390/vetsci9020041/s1, Table S1: summary of the publications that were included as eligible.

## Appendix A

**Table A1:** Preferred Reporting Items for Systematic reviews and Meta-Analyses extension for Scoping Reviews (PRISMA-ScR) Checklist.

Section	Item	Prisma-ScR Checklist Item	Reported on Page
Title			
Title	1	Identify the report as a scoping review.	1
Abstract			
Structured summary	2	Provide a structured summary that includes (as applicable): background, objectives, eligibility criteria, sources of evidence, charting methods, results, and conclusions that relate to the review questions and objectives.	1
Introduction			
Rationale	3	Describe the rationale for the review in the context of what is already known. Explain why the review questions/objectives lend themselves to a scoping review approach.	1–2
Objectives	4	Provide an explicit statement of the questions and objectives being addressed with reference to their key elements (e.g., population or participants, concepts, and context) or other relevant key elements used to conceptualize the review questions and/or objectives.	2
Methods			
Protocol and registration	5	Indicate whether a review protocol exists; state if and where it can be accessed (e.g., a Web address); and, if available, provide registration information, including the registration number.	n.a.
Eligibility criteria	6	Specify characteristics of the sources of evidence used as eligibility criteria (e.g., years considered, language, and publication status), and provide a rationale.	2
Information sources	7	Describe all information sources in the search (e.g., databases with dates of coverage and contact with authors to identify additional sources), as well as the date the most recent search was executed.	2
Search	8	Present the full electronic search strategy for at least one database, including any limits used, such that it could be repeated.	2
Selection of sources of evidence	9	State the process for selecting sources of evidence (i.e., screening and eligibility) included in the scoping review.	2–3
Data charting process	10	Describe the methods of charting data from the included sources of evidence (e.g., calibrated forms or forms that have been tested by the team before their use, and whether data charting was performed independently or in duplicate) and any processes for obtaining and confirming data from investigators.	3
Data items	11	List and define all variables for which data were sought and any assumptions and simplifications made.	n.a.
Critical appraisal of individual sources of evidence	12	If completed, provide a rationale for conducting a critical appraisal of included sources of evidence; describe the methods used and how this information was used in any data synthesis (if appropriate).	n.a.
Synthesis of results	13	Describe the methods of handling and summarizing the data that were charted.	3
Results			
Selection of sources of evidence	14	Give numbers of sources of evidence screened, assessed for eligibility, and included in the review, with reasons for exclusions at each stage, ideally using a flow diagram.	3
Characteristics of sources of evidence	15	For each source of evidence, present characteristics for which data were charted and provide the citations.	n.a.
Critical appraisal within sources of evidence	16	If completed, present data on critical appraisal of included sources of evidence (see item 12).	n.a.
Results of individual sources of evidence	17	For each included source of evidence, present the relevant data that were charted that relate to the review questions and objectives.	Suppl.mat.
Synthesis of results	18	Summarize and/or present the charting results as they relate to the review questions and objectives.	Suppl.mat.
Discussion			
Summary of evidence	19	Summarize the main results (including an overview of concepts, themes, and types of evidence available), link to the review questions and objectives, and consider the relevance to key groups.	8–11
Limitations	20	Discuss the limitations of the scoping review process.	9
Conclusions	21	Provide a general interpretation of the results with respect to the review questions and objectives, as well as potential implications and/or next steps.	11–12
Funding			
Funding	22	Describe sources of funding for the included sources of evidence, as well as sources of funding for the scoping review. Describe the role of the funders of the scoping review.	n.a.

JBI = Joanna Briggs Institute; PRISMA-ScR = Preferred Reporting Items for Systematic reviews and Meta-Analyses extension for Scoping Reviews.
