# Supplementary material for: Current Use of Equine Laparoscopy in Urogenital Disorders: A Scoping Review of the Literature from 2000 to 2021
Source: Vet Sci. 2022 Jan 22;9(2):41. doi: 10.3390/vetsci9020041 (PMC8876348; doi:10.3390/vetsci9020041)
Supplement: Supplementary file 1 [file vetsci-09-00041-s001.zip › vetsci-1531918-SI.pdf]

veterinary sciences

MDPI

Supplementary Material

Current Use of Equine Laparoscopy in Urogenital Disorders: A Scoping Review of the Literature from 2000 to 2021

Paola Straticò, Giulia Guerri, Adriana Palozzo, Vincenzo Varasano and Lucio Petrizzi

Table S1: Summary of the publications that were included as eligible.

Author (Year)	Country of Origin	Aim/Purpose	Study design	Target organ	Type of Surgery
**Adams and Hendrickson (2014) [19]**	USA	To review standing male urogenital surgery	Review	General	
**Al-Badrany (2007) [20]**	Iraq	To describe ovariectomy in donkeys with electrocautery and titanium clip	Original study	Ovary	Standing bilateral ovariectomy in donkeys
**Alford et al (2010) [21]**	USA	To describe laparoscopic and endoscopic NOTES for ovariectomy	Original study	Ovary	Standing surgery
**Allen et al (2006) [22]**	UK	To treat oviductal patency	Case series	Uterine tube	Standing laparoscopic topical application of a PGE_2 on the uterine tubes
**Alsafy (2013) [23]**	Egypt	Describe a novel electroligation ovariectomy	Original study	Ovary	Standing laparoscopic ovariectomy
**Arnold et al (2013) [24]**	USA	To evaluate oviductal patency	Original study	Uterine tube	Standing laparoscopic surgery
**Aziz et al (2008) [25]**	Iraq	To describe standing laparoscopic ovariectomy with a new instrument	Case series	Ovary	Standing laparoscopic ovariectomy in donkeys
**Bartel et al (2020) [26]**	The Netherlands	Surgical treatment of uterine leiomyoma	Case report	Ovary-Uterus	Standing hand-assisted ovariohysterectomy
**Bracamonte and Thomas (2017) [27]**	Canada	To document laparoscopic cryptorchidectomy with a vessel-sealing device	Restrospective	Testis	Dorsal recumbency laparoscopic cryptorchidectomy
**Brink et al (2010) [28]**	USA	To develop a laparoscopic technique of imbricating the mesometria to elevate the uterus	Case series	Uterus	Bilateral standing laparoscopic uteropexy
**Brommer et al (2011) [29]**	The Netherlands	Diagnosis of an abnormal cryptorchid testis	Case report	Testis	Standing laparoscopy
**Caron et al (2008) [30]**	USA	To report a technique for repair or prevention of inguinal hernia with intracorporeal suture closure of the internal inguinal and vaginal ring	Case series	Vaginal ring	Dorsal recumbency laparopic closure of the vaginal rings with intracorporeal sutures
**Carpenter et al (2006) [31]**	USA	Evaluation of the strength of different knots type	Original study	General effect of laparoscopy	Laboratory setting
**Clements et al (2019) [32]**	UK	To report the clinical findings in 10 horses diagnosed as monorchids	Case series	Testis	Standing laparoscopic or dorsal recumbency abdominal exploration
**Cockelaere et al (2005) [33]**	Belgium	To describe laparoscopic ovariectomy with a tie-rap	Original study	Ovary	Standing laparoscopic ovariectomy

**Cockelaere et al (2007) [34]**	Belgium	To describe the treatment of an unresolved case of ureteral ectopia in a filly	Case report	Kidney	Standing laparoscopic nephrectomy
**Colbath et al (2017) [35]**	USA	To describe a left flank approach for bilateral laparoscopic ovariectomy	Original study	Ovary	Standing laparoscopic ovariectomy
**Collar et al (2021) [36]**	USA	To describe behavioral changes after bilateral ovariectomy	Retrospective	Ovary	Standing laparoscopic ovariectomy
**Corsalini et al (2016) [37]**	Italy	to describe the surgical technique, complications and outcome of laparoscopic uteropexy in three mares using an absorbable barbed suture	Case series	Uterus	Standing laparoscopic uteropexy with intracorporeal barbed sutures
**Cribb and Bouré (2010) [38]**	USA	Laparoscopic management of a testicular teratoma	Case report	Testis	Standing laparoscopic cryptorchidectomy
**Cribb et al (2015) [39]**	USA	To compare laparoscopic and conventional open cryptorchidectomy in horses	Retrospective	Testis	
**Daniel et al (2015) [40]**	USA	Surgical management of an ovarian leiomyoma	Case report	Ovary	Standing ovariectomy
**Daniel et al (2015) [41]**	USA	Hand- assisted laparoscopic removal of large ovarian masses with and a sterile bag	Case series	Ovary	Standing hand-assisted ovariectomy
**DeBont et al (2010) [42]**	Belgium	To describe a plastic retrieval bag for removal of large dissected ovaries	Case series	Ovary	Standing laparoscopic ovariectomy
**Delcazo et al (2020) [43]**	Germany	Repair of a post-partum uterine tear with barbed sutures	Case report	Uterus	Standing left flank laparoscopic repair with intracorporeal barbed sutures
**Delcazo et al (2020) [44]**	Germany	Surgical treatment, complications and outcome of mares that underwent ovariohysterectomy	Original study	Ovary-uterus	First standing laparoscopic dieresis and hemostasis, second laparotomic hysterectomy
**De Fourmestraux et al (2014) [45]**	France	To evaluate the success rate of laparoscopic castration without orchidectomy	Original study	Testis	Standing laparoscopic dieresis and haemostasis without orchidectomy
**Delling et al (2004) [46]**	USA	To develop a minimally invasive, hand-assisted laparoscopic ovariohysterectomy	Original study	Ovary-uterus	Dorsal recumbency laparoscopic approach and hand assisted surgery through a hand-access device
**Desmaizieres et al (2003) [47]**	France	To report complications associated with cannula insertion	Retrospective	General effects of laparoscopy	Standing laparoscopy
**Devick et al (2018) [48]**	USA	To compare post-operative pain following active desufflation of CO_2 or no active desufflation after laparoscopic ovariectomies in mares	Original study	General effect of laparoscopy	Standing laparoscopic ovariectomy
**Devick and Hendrickson (2019) [49]**	USA	To evaluate the feasibility of a single flank approach for bilateral ovariectomy	Original study	Ovary	Standing laparoscopic ovariectomy

**Devick et al (2020) [50]**	USA	To assess efficacy of ovariectomy for unwanted behavior and to correlate it with endocrine profile	Retrospective	Ovary	Standing laparoscopic ovariectomy
**Diekstall et al (2018) [51]**	The Netherlands	Three methods for the treatment of post-partum uterine rupture	Original study	Uterus	Standing hand-assisted laparoscopic repair/dorsal recumbency laparoscopic repair with extracorporeal knots or intraabdominal barbed sutures
**Duesterdieck et al (2003) [52]**	USA	To evaluate use of the Harmonic Scalpel for laparoscopic bilateral ovariectomy	Original study	Ovary	Standing laparoscopic ovariectomy
**Duesterdiek-Zellmer (2007) [53]**	USA	Review of surgical treatment of urolithiasis	Review	Urinary bladder	
**Easley and Hendrickson (2014) [54]**	USA	Updates in techniques and instrumentation of equine laparoscopic surgery	Review	General	
**Easley et al (2017) [55]**	USA	To evaluate he temperature of a vessel sealer and divider device during unilateral paralumbar laparoscopic ovariectomy	Original study	Ovary	Standing laparoscopic ovariectomy
**El-Khamary and El-Sherif (2019) [56]**	Egypt	New laparoscopic approach and castration with two hemostasis techniques of normally descended testis	Original study	Testis	Lateral recumbency laparoscopic castration
**Farstvedt et al (2005) [57]**	USA	To compare mesovarian vs intraovaric local anaesthesia for laparoscopic ovariectomy	Original study	Ovary	Standing laparoscopic ovariectomy
**Finley and Fischer (2021) [58]**	USA	Removal of cryptorchid testicles in dorsally recumbent horses through an enlarged umbilical portal after laparoscopic intra-abdominal castration	Retrospective	Testis	Dorsal recumbency dieresis and haemostasis, umbilical laparotomy for testes removal
**Gablehouse et al (2009) [59]**	USA	To describe a new laparoscopic and vaginal management of ovariohysterectomy	Case report	Ovary-uterus	Standing laparoscopic dieresis and hemostasis of mesovarium and mesometrium, transvaginal uterine inversion and hysterectomy
**Goodin et al (2011) [60]**	USA	To review the efficacy and safety of unilateral ovariectomy by use of a standing hand-assisted laparoscopic approach and evaluate the effect of ovary size on postoperative complications	Retrospective	Ovary	Hand assisted standing laparoscopic ovariectomy
**Gracia-Calvo et al (2014) [61]**	Spain	Review of hernioplasty and herniorrhaphy of the inguinal rings in Equidae	Review	Vaginal ring	

**Gracia-Calvo et al (2015) [62]**	Spain	Assessment of testicular perfusion after peritoneal flap hernioplasty	Original study	Vaginal ring	Standing laparoscopic peritoneal flap hernioplasty and post-operative testicular pulsed-wave color Doppler ultrasound
**Graham and Freeman (2014) [63]**	USA	To describe standing abdominal surgical procedures	Review		
**Hand et al (2002) [64]**	USA	To evaluate a vessel-sealing instrument for hemostasis	Original study	Ovary	Standing laparoscopic ovariectomy
**Hanrath and Rodgerson (2002) [65]**	USA	To describe a laparoscopic cryptorchidectomy using electrosurgical instrumentation	Retrospective	Testis	Standing cryptorchidectomy
**Hartman et al (2015) [66]**	USA	To evaluate the management of equids undergoing cryptorchidectomy	Retrospective	Testis	Cryptorchidectomy
**Hendrickson (2006) [67]**	USA	To describe laparoscopic ovariectomy and cryptorchidectomy	Review	General (Ovary and testis)	
**Hendrickson (2009) [68]**	USA	Complications of laparoscopic surgery	Review	General	
**Hilton et al (2008) [69]**	USA	Surgical management of a renal cell carcinoma	Case report	Kidney	Hand-assisted laparoscopic nephrectomy
**Holmes et al (2012) [70]**	Australia	Laparoscopic cauterization of testicular arteries to manage haemoperitoneum	Case report	Testis	Standing laparoscopic surgery
**van Hoomoed and Galuppo (2005) [71]**	USA	To evaluate use of ENDO-GIA staples and ENDO-Catch pouches for ovariectomy in mares and to evaluate the efficacy of epidural morphine analgesia	Original study	Ovary	Standing laparoscopic ovariectomy
**Hubert et al (2006) [72]**	USA	To describe a laparoscopic removal of granulosa cell tumor with a vessel sealing device (LigaSure)	Retrospective	Ovary	Standing laparoscopic ovariectomy
**Huppes et al (2016) [73]**	The Netherlands	To review the efficacy of two protocol for decision making for cryptorchidism	Retrospective	Testis	
**Janicek et al (2004) [74]**	USA	Laparoscopic management of a uterine leiomyoma	Case report	Uterus	Standing hand-assisted laparoscopic removal of neoplasia
**Jones et al (2020) [75]**	USA	Ovariectomy as a treatment for pyometra	Case report	Ovary	Standing ovariectomy
**Joyce (2008) [76]**	USA	To compare pain responses during cryptorchidectomy following intratesticular or mesorchial infiltration of lidocaine	Original study	Testis	Standing cryptorchidectomy
**Joyce and Hendrickson (2008) [77]**	USA	To highlight the technique of laparoscopic cryptorchidectomy	Review	Testis	
**Kadic et al (2019) [78]**	Canada	A two-step laparoscopically assisted technique for	Case report	Ovary-uterus	Monolateral standing laparoscopic dieresis and hemostasis of mesovarium and mesometrium, dorsal

		ovariohysterectomy with a left-flank approach			recumbent laparotomic ovariohysterectomy
**Kelmer et al (2006) [79]**	USA	The diagnosis of monorchidism as another advantage of laparoscopic cryptorchidectomy in the horse	Case report	Testis	Standing laparoscopic surgery
**Keoughan et al (2003) [80]**	USA	To describe a hand-assisted, laparoscopic technique to remove the left kidney in standing horse	Case report	Kidney	Standing hand-assisted left nephrectomy
**Koch et al (2019) [81]**	USA	To compare pain-related responses with topical or injected anesthesia of the ovarian pedicle	Original study	Ovary	Monoliteral standing laparoscopic ovariectomy
**Köllmann et al (2011) [82]**	Germany	To develop a minimally invasive laparoscopic technique for catheterization of the infundibulum and orthograde flushing of the oviduct	Original study	Uterine tubes	Standing bilateral laparoscopic surgery
**Kummer et al (2010) [83]**	Switzerland	To describe a motorized morcellator technique for laparoscopic removal of granulosa-theca cell tumors (GCT)	Case series	Ovary	Standing laparoscopic ovariectomy
**Lacitignola et al (2020) [84]**	Italy	To describes the use of a commercial wound retractor laparoscopic system	Case report	Testis	Standing laparoscopic cryptorchidectomy
**Leonardi et al (2021) [85]**	Italy	To report a dentigerous teratoma of a retained testis	Case report	Testis	Standing laparoscopic cryptorchidectomy
**Lloyd et al (2007) [86]**	UK	To evaluate success of standing laparoscopic removal of enlarged ovaries with electrosurgical haemostasis	Retrospective	Ovary	Standing ovariectomy
**Loesch and Rodgerson (2003) [87]**	Florida	To describe advantages and disadvantages of existing techniques for ovariectomy	Review	Ovary	
**Lund et al (2013) [88]**	USA	To describe the removal of bladder urolith in the standing horse	Case report	Urinary bladder	Standing laparoscopic cystotomy and urolith removal with a customized retractable metal hook basket forceps
**Lund et al (2014) [89]**	USA	To describe the use of a motorized morcellator for elective bilateral laparoscopic ovariectomy	Case series	Ovary	Standing laparoscopic ovariectomy
**Marien (2001) [90]**		Cylindrical mesh herniorrhaphy in 9 stallions	Case series	Vaginal ring	Standing laparoscopic herniorrhaphy
**Major et al (2017) [91]**	USA	To compare laparoscopic single-layer versus double-layer closure of incisions in adult urinary bladders with uni- and	Original study	Urinary bladder	Ex-vivo

		bidirectional barbed suture			
**McNally et al (2012) [92]**	USA	Surgical management of a chronic uterine tear	Case report	Uterus	Hand-assisted standing laparoscopic repair of a uterine tear
**Merchan et al (2021) [93]**	USA	To describe a hand-assisted laparoscopic removal of cystic calculi in male horses	Case report	Urinary bladder	Dorsal recumbency laparoscopic cystotomy and hand-assisted removal of bladder urolith
**Muurlink et al (2008) [94]**	UK	Removal of a uterine leiomyoma	Case report	Uterus	Standing laparoscopic approach to the uterine horn
**Oreff et al (2015) [95]**	Israel	Management of urethral injury with perineal urethroplasty and laparoscopic-assisted tube cystotomy	Case report	Urinary bladder	Laparoscopic-assisted tube cystotomy
**Pader et al (2011) [96]**	USA	To develop and assess the feasibility of (NOTES) ovariectomy in the mare.	Original study	Ovary	Standing NOTES ovariectomy
**Pader et al (2011) [97]**	USA	To compare NOTES ovariectomy and laparoscopic ovariectomy	Original study	Ovary	Standing NOTES ovariectomy
**Peer et al (2012) [98]**	Germany	Removal of internal gonad in two intersex horses	Case report	Testis	Standing laparoscopic removal of internal gonad
**Pepe et al (2005) [99]**	Italy	To describe a laparoscopic sterilization of the male donkey using an endoscopic linear stapler	Case series	Testis	Standing laparoscopic castration with endoscopic stapler
**Petrizzi et al (2020) [100]**	Italy	To describe laparoscopic ovariectomy in standing mules, abdominal anatomy, and resolution of unwanted behavior	Case series	Ovary	Standing laparoscopic ovariectomy
**Pye et al (2018) [101]**	Wales	To describe the laparoscopic transection of restrictive bands of the mesosalpinx and topical application of prostaglandin E_2 to treat suspected uterine tubal blockage	Original study	Uterine tube	Bilateral standing laparoscopic resection of fibrous band and topical application of PGE_2
**Quéré et al (2019) [102]**	France	Laparoscopic management of a uterine adenocarcinoma	Case report	Uterus	Standing hand-assisted laparoscopic removal of neoplasia
**Ragle et al (2013) [103]**	USA	To describe a unidirectional barbed suture for closure of the vaginal ring as a testicle-sparing technique	Case report	Vaginal rings	Standing laparoscopic closure of the vaginal ring with a unidirectional barbed suture
**Rambags et al (2003) [104]**	The Netherlands	Removal of GCTs with significant adhesions to important abdominal organs	Case series	Ovary	Exploratory laparoscopy and laparotomic ovariectomy and adhesiolysis
**Randleff-Rasmussern et al (2016) [105]**	South Africa	To describe the surgical management of a metallic foreign body withing the ovary	Case report	Ovary	Standing ovariectomy
**Rijkenhuizen (2002) [106]**	The Netherlands	Trreatment of post-operative haemor-rhages in two castrated stallions.	Case report	Testis	Standing laparoscopic surgery

**Rijkenhuizen and van Dijk (2002) [107]**	The Netherlands	To report diagnostic and therapeutic applications of laparoscopy	Retrospective	General	
**Rijkenhuizen and Goehring (2003) [108]**	The Netherlands	Laparoscopic repair of a bladder rupture	Case report	Urinary bladder	Dorsal recumbency laparoscopy
**Rijkenhuizen et al (2008) [109]**	The Netherlands	Repair of a bladder rupture in an adult mare during parturition	Case report	Urinary bladder	Dorsal recumbency laparoscopic approach
**Rijkenhuizen and van der Harst (2017) [110]**	The Netherlands	To report a laparoscopic-assisted abdominal castration descended testes in unilateral abdominal cryptorchids	Case series	Testis	Standing laparoscopic assisted castration of descended testes d
**Rijkenhuizen et al (2018) [111]**	The Netherlands	Removal of abdominal cystic testicles and outcome in two male horses	Case report	Testis	Standing laparoscopic cryptorchidectomy
**Rijkenhuizen et al (2021) [112]**	The Netherlands	Laparoscopic ligation of the mesovarium without removal of the ovaries	Original study	Ovary	Standing laparoscopic ovariectomy
**Rocken et al (2006) [113]**	Germany	To describe a technique for laparoscopic-assisted removal of cystic calculi in geldings and report outcome	Case series	Urinary bladder	Dorsal recumbency laparoscopic-assisted cystotomy
**Rocken et al (2007) [114]**	Germany	To describe technique and outcome of laparoscopic-assisted nephrectomy in case of unilateral renal disease.	Case report	Kidney	Standing hand-assisted laparoscopic nephrectomy
**Rocken et al (2011) [115]**	Germany	To evaluate surgical techniques, perioperative complications and outcome unilateral and bilateral laparoscopic ovariectomy	Retrospective	Ovary	Standing laparoscopic ovariectomy
**Rodgerson et al (2001) [116]**	USA	To describe in horses and ponies a laparoscopic ovariectomy by electrosurgical instrumentation	Case series	Ovary	Standing laparoscopic ovariectomy with electrosurgical instrumentation in horses, dorsal recumbency in ponies
**Rodgerson et al (2002) [117]**	USA	To describe a hand-assisted laparoscopic ovariectomy technique to remove enlarged ovaries in standing mares	Retrospective	Ovary	Hand-assisted standing laparoscopic ovariectomy
**Roessner et al (2015) [118]**	USA	To investigate the predictive effect of altrenogest on bilateral ovariectomy to treat behavioral problems	Retrospective	Ovary	Standing laparoscopic ovariectomy
**Romero et al (2010) [119]**	Spain	To report surgical management of a nephroblastoma	Case report	Kidney	Standing hand-assisted laparoscopic nephrectomy
**Rossignol et al (2007) [120]**	France	To evaluate the efficacy of a laparoscopic peritoneal flap hernioplasty (PFH)	Original study	Vaginal rings	Standing laparoscopic herniorrhaphy
**Rossignol et al (2014) [121]**	France/Germany	Hernioplasty in standing stallions using cyanoacrylate glue	Case series	Vaginal rings	Standing laparoscopy
**Rubiomartinez (2011) [122]**	South Africa	Standing laparoscopic surgical anatomy and removal of the internal	Case report	Testis	Standing laparoscopic surgery

		gonads in an equine male pseudohermaphrodite			
**Ruzickova et al (2016) [123]**	Canada	To compare a knotless, barbed suture to standard suture in an ex vivo model	Original study	Urinary bladder	Ex vivo
**Sassot et al (2017) [124]**	USA	To describe the use of a motorized morcellator for extraction of abdominal testes *via* an existing portal	Case series	Testis	Standing laparoscopic cryptorchidectomy
**Shoemaker et al (2004) [125]**	Canada	To assess the feasibility of laparoscopic ovariectomy without removal of the ovaries from the abdomen of juvenile horses	Original study	Ovary	Dorsal recumbency laparoscopic ovariectomy
**Seabaugh et al (2013) [126]**	USA	To compare effect between 2 methods for cryptorchid testis vessel hemostasis on peritoneal fluid values	Original study	Testis	Standig laparoscopic cryptorchidectomy
**Seabaugh and Schumacher (2014) [127]**	USA	To review standing urogenital surgery	Review	General	
**Seabaugh et al (2014) [128]**	USA	To compare peritoneal fluid characteristics after laparoscopic ovariectomy with two hemostatic methods	Original study	Ovary	Standing laparoscopic ovariectomy
**Serena et al (2009) [4]**	USA	To describe surgical management and outcome of resection of a squamous cell carcinoma	Case report	Urinary bladder	Dorsal recumbency laparoscopic assisted cystotomy
**Silva et al (2008) [129]**	Brasil	Overview of surgical procedures and their applications	Review	General	
**Smith et al (2005) [130]**	UK	To review updates in equine abdominal surgery	Review	General	
**Smith and Mair (2008) [131]**	UK	To report the complication rates associated with standing laparoscopic ovariectomy by sequential electro-coagulation and sharp transection	Case series	Ovary	Standing laparoscopic ovariectomy
**Smith and Devine (2019) [132]**	UK	To describe the laparoscopic diagnosis and castration of a monorchid male pseuohemraphrodite	Case report	Testis	Dorsal recumbency laparoscopic assisted castration
**Smith and Devine (2013) [133]**	UK	To determine the feasibility of hand-assisted laparoscopic ovariectomy via colpotomy for bilateral ovariectomy in standing mares	Case series	Ovary	Hand-assisted laparoscopic ovariectomy standing mares.
**Straticò et al (2012) [134]**	Italy	To report laparoscopic-assisted cystotomy and inguinal cystostomy for treating bladder urolithiasis in a gelding	Case report	Urinary bladder	Dorsal recumbency laparoscopic assisted cystotomy
**Straticò et al (2020) [135]**	Italy	To review characteristics of patients referred for cryptorchidism	Retrospective	Testis	
**Tate et al (2012) [136]**	USA	To describe an ovariectomy through a colpotomy with a chain ecraseur	Original study	Ovary	Ovariectomy via colpotomy under laparoscopic guidance

**Tuohy et al (2009) [137]**	USA	Repair of bladder rupture in a stallion	Case report	Urinary bladder	Standing laparoscopic approach
**Vanhaesebrouck et al (2010) [138]**	Belgium	To describe surgical treatment of an ovarian teratoma	Case report	ovary	Standing laparoscopic ovariectomy
**Velez et al (2012) [139]**	USA	To evaluate the health and ovarian status of mares subjected to repeated TVAs for collection of oocytes	Original study	Ovary	Standing laparoscopy
**Vitoria et al (2019) [140]**	Spain	To describe a standing laparoscopic vasectomy in stallions and its outcome	Case series	*Ductus deferens*	Standing laparoscopic vasectomy with a laparoscopic vessel sealing device
**Vitte et al (2013) [141]**	France	Laparoscopic-assisted removal of cystic calculi thought the inguinal canal in intact males	Case series	Urinary bladder	Dorsal recumbency laparoscopic and NOTES through the inguinal canal
**Vitte et al (2014) [142]**	France	To report a combination of laparoscopic dissection of the mesovarian pedicle and a celiotomy under general anesthesia for removal of the ovary	Original study	Ovary	Standing laparoscopic dieresis and hemostasis, dorsal recumbency laparotomic removal
**Voermans et al (2006) [143]**	The Netherlands	To establish the prevalence of testicular revascularization in case of laparoscopic castration without cryptorchidectomy	Original study	Testis	Standing and dorsal recumbency laparoscopic castration without orchiectomy
**Waguespack et al (2001) [144]**	USA	To describe laparoscopic management of post castration haemorrage	Case report	Testis	Standing laparoscopic haemostasis
**Walesby et al (2002) [145]**	USA	To describe the surgical management of a urinary bladder rupture in an adult horse	Case report	Urinary bladder	Dorsal recumbency laparoscopy
**Wilderjans et al (2012) [146]**	Belgium	Describe laparoscopic peritoneal flap hernioplasty (PFH)	Case series	Vaginal ring	Standing laparoscopy
**Wilderjans et al (2021) [147]**	Belgium	To describe a minimally invasive laparoscopic tacked intra-peritoneal slitted mesh (TISM) technique to close the vaginal rings	Original study	Vaginal rings	Bilateral standing laparoscopic closure of vaginal rings
**Woodford et al (2014) [149]**	UK	To describe the surgical management of pyometra in 3 mares	Case report	Ovary-uterus	Standing laparoscopic dissection and haemostasis, dorsal recumbency laparotomic removal of ovaries and uterus
